# Removal of lead from aqueous solutions using three biosorbents of aquatic origin with the emphasis on the affective factors

**DOI:** 10.1038/s41598-021-04744-0

**Published:** 2022-01-14

**Authors:** Maryam Rezaei, Nima Pourang, Ali Mashinchian Moradi

**Affiliations:** 1 Department of Marine Biology, Science and Research Branch, Islamic Azad University, Tehran, Iran; 2 Iranian Fisheries Science Research Institute (IFSRI), Agricultural Research, Education and Extension Organization (AREEO), Tehran, Iran

**Keywords:** Environmental sciences, Ocean sciences

## Abstract

The biosorptive potentials of three aquatics-based biosorbents, including shells of a bivalve mollusk and scales of two fish species for Pb removal from aqueous solutions were evaluated, for the first time. A Box–Behnken design with the response surface methodology was used to investigate the effects of the seven important variables (contact time, temperature, initial concentration, dosage, size, salinity and pH) on the sorption capacity of the sorbents. Among the seven studied factors, the effects of biosorbent dosage, initial concentration and pH were significant for all the response variables, while biosorbent size was not significant for any of the responses. The initial concentration was the most influential factor. The presence of Pb ions on the surfaces of the biosorbents after the adsorption was clearly confirmed by the SEM–EDX and XRF analyses. The maximum sorption capacities of the biosorbents were comparable to the literature and the descending order was as follows: scales of *Rutilus kutum* and *Oncorhynchus mykiss* and the shells of *Cerastoderma glaucum*. The isotherm studies revealed Langmuir model applicability for the Pb adsorption by *R. kutum* and *O. mykiss scales*, while Freundlich model was fitted to the adsorption *C. glaucum* shells.

## Introduction

Pollution of aquatic ecosystems caused by heavy metals has been one of the major environmental threats over the last several decades and is of high ecological significance. These concerns are arise from their non-biodegradability, high toxicity and huge discharge into the environment^1^. Heavy metals occur naturally in aquatic ecosystems, but with large variations in concentration. They also enter the environment from various man-made sources. These metals are released into the aquatic environments through direct discharges into both freshwater and marine ecosystems or through indirect routes^2, 3^. These hazardous pollutants tend to transfer through the food chains and potentially can cause adverse effects on the health of any organisms at any trophic level. Hence, the removal of heavy metal from contaminated waters has become one of the most imminent environmental problems^4, 5^.

Pb(II) is classified as a non-essential prevalent toxic metal ions and major environmental health problems, which affects multiple body systems including the hematologic, neurologic, gastrointestinal, renal and cardiovascular systems^6, 7^. Lead in the form of Pb(II) is one of the most stable and toxic ions in aquatic ecosystems and shows considerable tendency to accumulate in various organs of aquatic organism^8, 9^. The major sources of Pb(II) in aquatic ecosystem are anthropogenic, including municipal wastewater and industrial effluents discharged from different industries manufacturing batteries, pigments, cables, pipes, ceramics, gasoline, tobacco, steel, food packaging glasses and pesticides^10, 11^. Annually, large quantities of heavy metals, including lead (derived from various urban, agricultural and industrial sources), enter the Southern Caspian Sea either directly or through rivers^12, 13^.

There are some widely used methods for removal of Pb(II) and other heavy metal ions from wastewater, such as membrane filtration, electrolytic recovery, precipitation, ion exchange, adsorption and so on; however, these conventional methods can cause some important problems such as management of generated wastes, production of toxic sludge that require safe disposal and high cost^14, 15^. In the past three decades, there has been a growing interest in developing low cost and environment friendly materials for removal of heavy metals from wastewater and natural environment^16^.

Adsorption is a highly effective and economic separation and purification method that is increasingly being utilized for the removal of heavy metals ions from industrial effluents^17–19^. Up to now, a wide variety of natural and synthetic adsorptive materials, such as natural materials, industrial byproducts, forest and agricultural wastes, biopolymers, compost and nanomaterials, have been studied and some of them are currently employing extensively for the removal of different metal ions^20–23^.

Biosorption is an emerging and promising technology for the removal of toxic metals from industrial effluents and natural waters^24^. The biosorption process utilizes the ability of nonliving biomaterials to eliminate heavy metals from wastewater effluents using metabolically mediated or physico-chemical pathway of uptake, and is based on different mechanisms (e.g. absorption, adsorption, surface complexation, ion exchange and precipitation)^11, 25, 26^.

A wide range of biosorbents have been applied to remove heavy metal ions, including Pb(II), from aqueous solutions. Among them, according to the relatively limited but promising previous studies, two types of aquatic origin biosorbents have been found to have good potential in this regard, which include fish scales^4, 27–32^ and mollusk shells^1, 5, 15, 33–36^. Fish scales are consist mainly of collagen fibers and hydroxyapatite. The structure of collagen shows that it contains the possible functional groups, which are involved in the biosorption of heavy metals. On the other hand, the porous structure of hydroxyapatite has relatively high adsorption capacity^37, 38^. The studies on application of bivalve mollusk shells have demonstrated that these naturally occurring, inexpensive and plentiful materials, which are very common around the coasts of many coastal countries, can be considered as potential cost-effective biosorbents^39, 40^.

The main objective of this research was to investigate the removal potential of lead (II) from aqueous solutions by using three aquatic origin biosorbents, i.e. scales of two fish species (*Rutilus kutum* and *Oncorhynchus mykiss*) and shells of a bivalve mollusk (*Cerastoderma glaucum*). The influences of some important parameters, i.e. sorbent dosage, sorbent size, contact time, temperature, initial concentration, pH and salinity) on the sorption capacity of the biosorbents were also evaluated. The three selected biosorbents are environmentally friendly, economically feasible and abundant. *Rutilus kutum* is the most important commercial bony fish in southern part of the Caspian Sea^41^. *Oncorhynchus mykiss* is the leading freshwater farmed species in Iran^42^. *Cerastoderma glaucum* has a relatively wide distribution along the Iranian coast of the Caspian Sea^43^. All the three studied sorbents are easily accessible in large quantities at a very low cost. Rainbow trout, *O. mykiss* is the main cold water cultured fish species in Iran^44^. Caspian Kutum, *R. kutum* is the most commercially important bony fish at the southern Caspian Sea^41^. *Cerastoderma glaucum* is one of the dominant bivalve species in the southern Caspian Sea and its dead shells can be collected easily, inexpensively and abundantly along the coast^45, 46^.

It is noticeable that, so far, no report is available on the use of the three investigated biomaterials as biosorbents for the removal of heavy metals or other pollutants from contaminated waters. Moreover, the simultaneous effects of seven important parameters on the adsorbance efficiency of the selected biosorbents have also been evaluated. Internationally, no other similar research has been conducted in this regard.

## Materials and methods

### Sample collection and preparation

**Figure 1:**
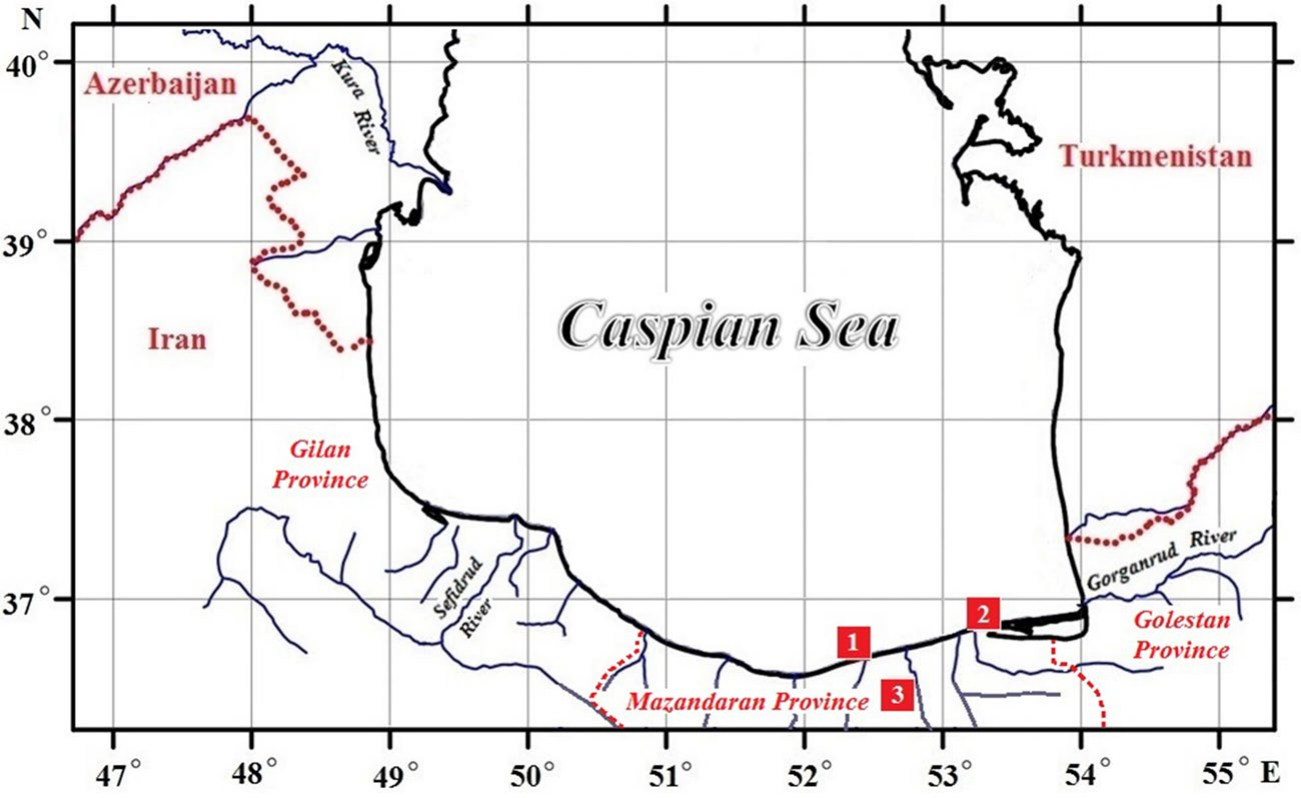
Map of the Caspian Sea showing the locations of collection of the specimens. 1: *Rutilus kutum*, 2: *Cerastoderma glaucum*, 3: *Oncorhynchus mykiss*. Map created by the authors using ArcGIS 10.7 (https://desktop.arcgis.com/en/).

Shells of *C. glaucum* (about 2 kg) were collected from the beaches of Miankaleh area (southeastern Caspian Sea). Fifty rainbow trout specimens were obtained from a fish farm located in Mazandaran Province. Fifty *R. kutum* specimens were also randomly collected from commercial beach seine catches in the coastal area (Fig. 1). The scales of the specimens were taken from above the lateral line. The shells and scales samples were transferred into plastic containers and taken to the laboratory. The scales and shells were washed several times with fresh running water to eliminate any adhering dirt and debris, and then soaked in double distilled water for 24 h, and later rinsed three times with double distilled water. The scales and shell samples dried at sunlight for two days. They were dried thereafter in oven at 60 °C until a constant weight. The dried shell fragments were then ground using a mortar and pestle and the scales pulverized in a laboratory blender. The biosorbents screened through a set of standard nylon sieves to obtain the desired particle sizes (0.4, 0.7 and 1.0 mm). The samples were then preserved in clean air-tight polyethylene containers for the further use^35, 47, 48^.

### Metal solution preparation

The Pb stock solution was prepared by dissolving 1.598 g of lead (II) nitrate (Merck, Germany) in 100 mL of ultrapure water, diluted with deionized water up to 1000 mL. The working solutions (30, 65 and 100 mg/L) were prepared by diluting the stock solution with double distilled water. The pH of the solutions was adjusted by the addition of 0.1 M HCl or NaOH. All chemicals used in the present study were of analytical grade^11, 15^.

### Biosorbents characterization

The morphology of the biosorbents before and after Pb(II) ion adsorption as well as the chemical composition were analyzed by Scanning Electron Microscopy (SEM) (SNE-4500 M, SEC, South Korea) coupled with Energy Dispersive X-ray analysis (EDX). Before the analyses, the samples were mounted onto aluminum stubs using double-sided carbon tape, gold coated with a sputter coater. The accelerating voltage for SEM imaging was 20 kV^49^. The biosorbents were also analyzed before and after Pb adsorption for surface functional groups using Fourier Transform Infrared Spectrometer (FTIR) (Agilent Cary 630 FTIR, Agilent Technologies, USA). The analysis was conducted using potassium bromide pellets as a reference material. The samples were examined in the range 650–4000 cm^−1^^6, 35, 48^. Moreover, X-Ray Fluorescence (XRF) (PW 1480, Philips, Netherlands) was used to determine the chemical composition of the biosorbents before and after the Pb adsorption^28, 50^.

### Adsorption isotherms

Biosorption isotherms were evaluated by varying the initial metal ion concentrations from 30 to 170 mg/L. In the isotherm experiments, with regards to the results concerning effects of the independent variables on the responses, the values of other independent variables (i.e. biosorbent dosage, contact time, temperature, pH and salinity) remained constant. In order to diagnose the nature of adsorption (homogeneous or heterogeneous) onto the biosorbents, four theoretical isotherm models, namely Langmuir, Freundlich, Temkin and Dubinin-Radushkevich were used.

### Experimental design and data analysis

Box–Behnken experimental design (BBD) combined with response surface method (RSM) was employed to assess and optimize the effects of selected variables on the responses. Each of the independent variables included three levels coded as − 1, 0, and + 1 for low, average, and high values, respectively. The experimental ranges of the independent variables were as follows: initial concentration (30–100 ppm), biosorbent dosage (0.1–0.3 g/L), biosorbent size (0.4–1.0 mm), contact time (2–5 h), pH (5.5–7), salinity (0.2–10 ppt) and temperature (20–30 °C). A BBD with 7 factors, 3 levels and 62 runs (with 6 replicates at center point) was used for the optimization of the three responses (PbR: concentration of Pb adsorbed by scales of *Rutilus kutum*; PbO: concentration of Pb adsorbed by scales of *Oncorhynchus mykiss* and PbC: concentration of Pb adsorbed by shells of *Cerastoderma glaucum*). Design-expert software (version 11) was utilized to analyze the experimental data. The statistical validation was performed by assessment of statistical parameters such as model F-value, lack of fit F-value, coefficient of determination (R^2^), adjusted R-squared (R^2^_Adj_), predicted R-squared (R^2^_Pred_), predicted residual error sum of squares (PRESS) and adequate precision (AP). Analysis of variance (ANOVA) was applied to evaluate the statistical significance and adequacy of the model (one test for each response variable)^51^. By constructing a normal probability plot of the residuals, the normality assumption of each ANOVA was checked. Subsequently, the response variables were transformed to achieve normality using Box-Cox procedure (natural logarithmic transformation for all the responses)^52, 53^.

### Biosorption studies and metal analysis

The biosorption experiments were carried out discontinuously in Erlenmeyer flasks (250 mL). The sample size in the Erlenmeyer was 100 mL. The prepared solutions were shaken at 180 rpm using a vibratory shaker. The experiments were done by varying the seven independent variables according to the Box-Behnken experimental design mentioned previously. The desired pH of the solutions was maintained by adding 1 mol/L HCl or NaOH at the beginning of the experiment. All the experiments were performed in triplicates and the mean values were presented. After the biosorption process, the biosorbents were separated from the aqueous solution by filtering using Watchman No.1 filter paper. The biosorbents samples were prepared for Pb concentration analysis in accordance with the MOOPAM Instruction^54^. Atomic absorption spectrophotometer (AAS) (SOLAAR M5, Thermo Electron Corp., Verona, WI, USA) was employed to measure the concentrations of lead in the biosorbents before and after the process. The difference between these two was considered as the concentration of Pb adsorbed by the biosorbents. The detection limit (calculated on the basis of ten determinations of the blanks as three times the standard deviations of the blank) was 0.05 ppm. The analytical accuracy and precision were verified using standard reference materials (SRMs) (oyster tissue: NIST 1566 b; tuna fish flesh: IAEA-436). The recovery rates were in the range of 96.4–102.6%. The precision of the analyses were estimated by calculating the relative standard deviation (RSD/%) based on replicate analyses (n = 10) of the SRMs. The precision was less than three percent RSD for all the determinations.

## Results

### Statistical analysis and models

As mentioned previously, in order to measure how well the suggested model fits the experimental data, different statistical parameters were evaluated. The resulting models in the present research were tested by ANOVA. Table 1 shows the ANOVA results for all the three responses. According to the results presented in the table, F-ratios of the models were 53.29, 27.90 and 26.57, while their lack-of-fits were 1.46, 0.64 and 4.46 for PbR, PbO and PbC, respectively. The low probability value of the models (*p* < 0.0001) and non-significant (*p* > 0.05) lack of fits indicate that all the three models are highly significant and valid for the present work. The quadratic polynomial models (except for PbC, which was linear) representing the relationship between the response variables and the chosen factors were generated using the values of the experimental data and given below (Eqs. 1, 2 and 3):

1 $$\begin{aligned} \ln \left( {PbR} \right) \, & = \, 7.28 - 0.7235{\text{A}} + 0.1768{\text{C}} + 0.0611{\text{D + }}1.07{\text{E}} - 0.6499{\text{F}} - 0.2312{\text{G}} \\ & \quad - 0.201{\text{AF}} - 0.4351{\text{DE}} - 0.1902{\text{FG}} - 0.4525{\text{A}}^{2} - 0.1315{\text{C}}^{2} - 0.2704{\text{E}}^{2} \\ \end{aligned}$$

2 $$\begin{aligned} {\text{ln}}\left( {{\text{PbO}}} \right) \, & = { 6}.{65 } - \, 0.{\text{5914A }} - \, 0.00{\text{48B }} - \, 0.0{\text{659C }} + { 1}.0{\text{9E }} - \, 0.{\text{4972F }} - \, 0.{149}0{\text{G}} \\ & \quad - 0.{\text{3333EG }} + \, 0.{34}0{\text{6A}}^{2} \, + \, 0.{\text{5451B}}^{2} + 0.{\text{4213C}}^{2} + 0.{\text{2844E}}^{2} \\ \end{aligned}$$

3 $${\text{ln}}\left( {{\text{PbC}}} \right) \, = { 6}.{14 } + \, 0.{\text{1481A }} - \, 0.0{\text{151B }} + \, 0.0{\text{168C }} - \, 0.{\text{1239D }} + \, 0.{\text{6585E }} + \, 0.{\text{3731F }} - 0.{618}0{\text{G}}$$

where A = Sorbent dosage, B = Sorbent size, C = Contact time, D = Temperature, E = Initial concentration, F = pH and G = Salinity.

**Table 1:** Analysis of variance summary of all responses (Y_1_–Y_3_) for the three fitted polynomial models.

Source	Y_1_(PbR)		Y_3_(PbO)		Y_3_(PbC)	
	F-ratio	*p*-value	F-ratio	*p*-value	F- ratio	*p*-value
Model	53.29	< 0.0001	27.90	< 0.0001	26.57	< 0.0001
A (Biosorbent Dosage)	138.31	< 0.0001	49.62	< 0.0001	6.41	0.0172
B (Biosorbent Size)			0.0032	0.9550	0.0630	0.8036
C (Contact Time)	8.26	0.0060	0.6169	0.4359	0.0764	0.7843
D (Temperature)	0.9873	0.3253			5.27	0.0294
E (Initial Concentration)	301.13	< 0.0001	169.00	< 0.0001	75.71	< 0.0001
F (pH)	111.61	< 0.0001	35.07	< 0.0001	22.80	< 0.0001
G (Salinity)	14.12	0.0005	3.15	0.0820	90.12	< 0.0001
AF	3.56	0.0652				
DE	16.67	0.0002				
EG			5.25	0.0261		
FG	3.19	0.0805				
A^2^	32.61	< 0.0001	9.81	0.0029		
B^2^			25.12	< 0.0001		
C^2^	2.76	0.1033	15.00	0.0003		
E^2^	11.64	0.0013	6.84	0.0118		
Lack of Fit	1.46	0.3462	0.64	0.6821	4.46	0.0517

PbR: Concentration of Pb adsorbed by scales of *Rutilus kutum*; PbO: Concentration of Pb adsorbed by scales of *Oncorhynchus mykiss*; PbC: Concentration of Pb adsorbed by shells of *Cerastoderma glaucum.*

These equations can be used to predict the responses for given levels of individual factors.

Table 2 lists the parameters used to fit the polynomial models.

**Table 2:** SD, Mean, CV, PRESS, AP and R^2^ for all the response variables.

	Y_1_(PbR)	Y_2_(PbO)	Y_3_(PbC)
Mean	7.61	7.26	6.18
SD	0.30	0.41	0.21
CV (%)	3.96	5.66	3.50
PRESS	7.83	13.70	1.90
R^2^	0.93	0.86	0.87
Adjusted R^2^	0.91	0.83	0.84
Predicted R^2^	0.87	0.80	0.81
AP	31.63	20.92	22.48

PbR: Concentration of Pb adsorbed by scales of *Rutilus kutum* ; PbO: Concentration of Pb adsorbed by scales of *Oncorhynchus mykiss*; PbC: Concentration of Pb adsorbed by shells of *Cerastoderma glaucum.*

**Figure 2:**
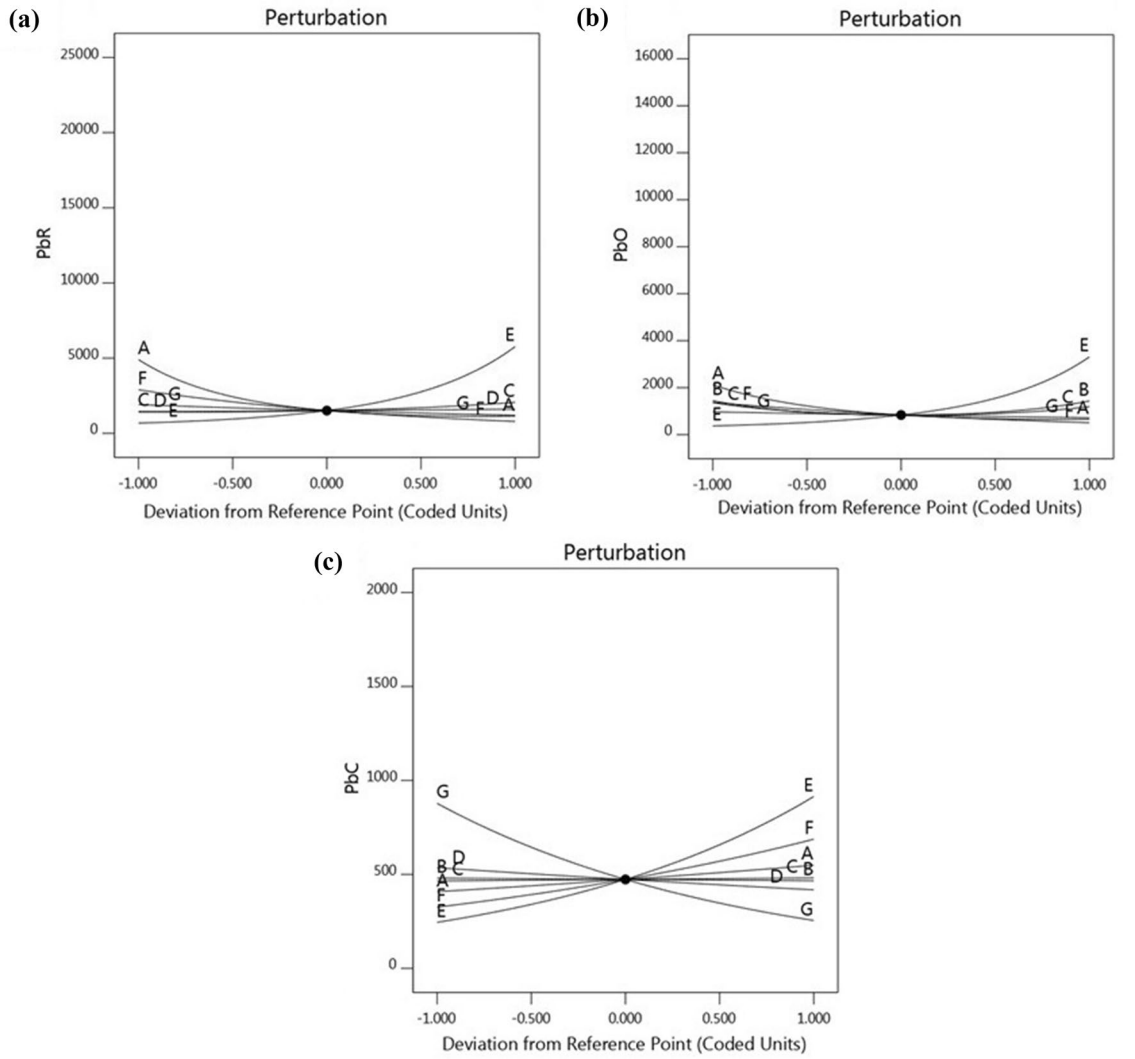
Perturbation plots showing the effect of all factors (A: biosorbent dosage, B: biosorbent size, C: contact time, D: temperature, E: initial concentration, F: pH and G: salinity) on the response variables (**a**: PbR, **b**: PbO, **c**: PbC).

In order to investigate the relative effects of each of the independent variables on the response variables (strength and direction of the effects), the perturbation plots were used, which are presented in Fig. 2.

**Figure 3:**
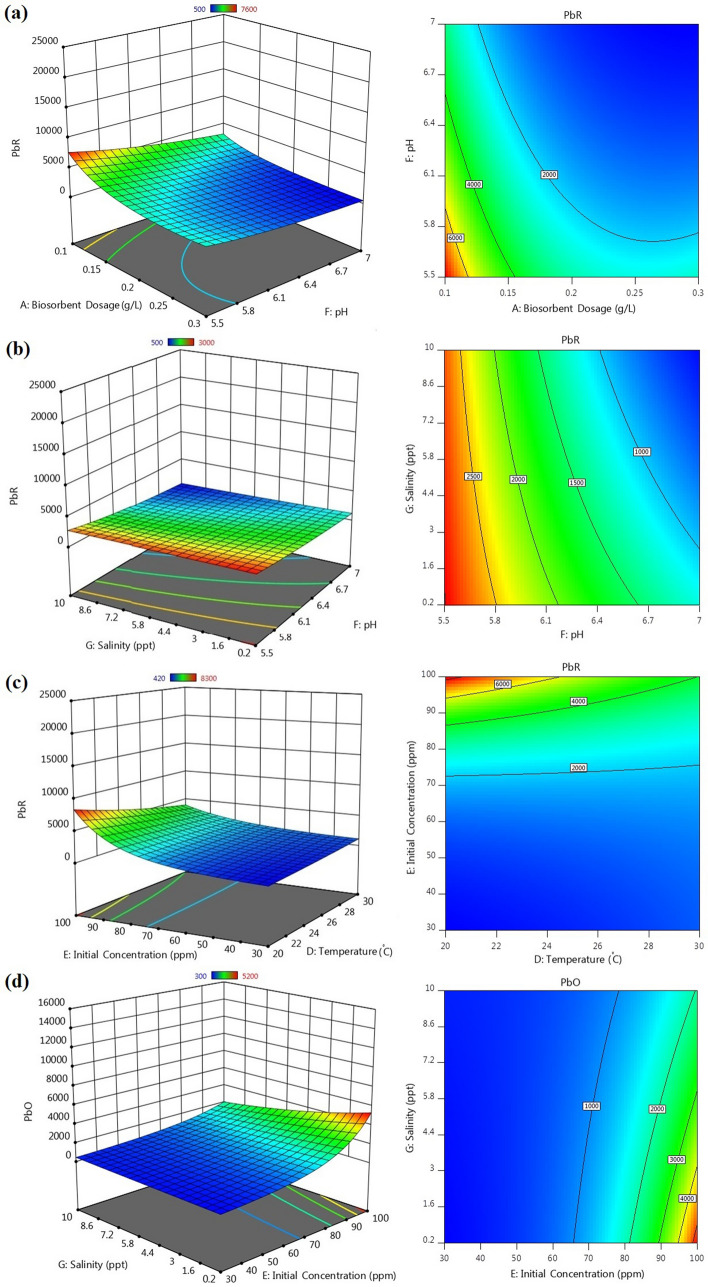
3D response surface plots and contour curves showing the interactive effects of (**a**) pH and biosorbent dosage on PbR, (**b**) pH and salinity on PbR, (**c**) temperature and initial concentration on PbR, (**d**) initial concentration and salinity on PbO.

In order to evaluate the interaction of factors on the response variables, response surface and contour plots were drawn (Fig. 3). As can be seen, each plot indicates the simultaneous effects of two independent variables within their investigated ranges, on the response, while keeping the other factors constant, generally at central point.

### Structural characteristics of the sorbents

**Figure 4:**
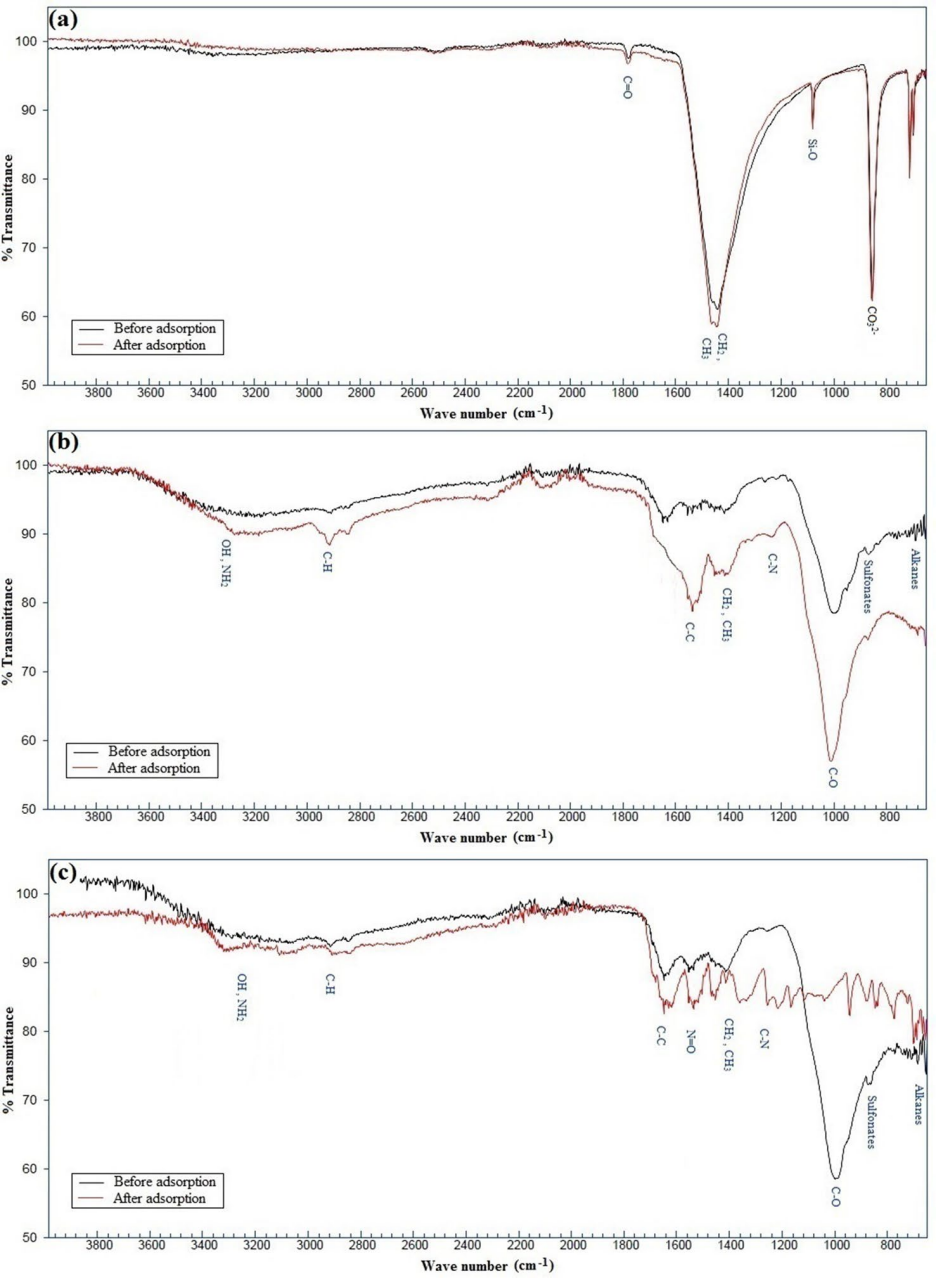
FTIR spectra of *C. glaucum* shell (**a**), *O. mykiss* scale (**b**), and *R. kutum* scale (**c**) before and after Pb adsorption indicating corresponding functional groups.

The FTIR spectra were used to determine the frequency changes in the functional groups existing on the surfaces of the biosorbents, before and after the Pb adsorption. Figure 4 shows the approximate positions of the absorbance peaks for the corresponding functional groups. Table 3 summarizes the assignments for the corresponding functional groups before and after the adsorption. As shown in the figure and Table, in case of *C. glaucum* shell, there is a shift in the calcite group from 854.814 to 856.134 cm^−1^, Si–O group from 1082.454 to 1082.789 cm^−1^, CH_2_ and CH_3_ groups from 1445.946 to 1449.595 cm^−1^, while the C=O group shifts from 1785.548 to 1786.826 cm^−11^. In the case of the other two studied sorbents, some shifts and disappearances of the peaks correspond to different functional groups can also be deduced from Fig. 4 and Table 3 (i.e. alkanes, sulfonates, C–O, C–N, CH_2_, CH_3,_ C–H for *O. mykiss* scale and alkanes, sulfonates, C–O, C–N, CH_2_, CH_3_, N=O, C–C, C–H, OH, NH_2_ for *R. kutum* scale).

**Table 3:** Wave number of dominant peaks obtained from FTIR transmission spectra of the three biosorbents (O: *O. mykiss* scales; R: *R. kutum* scales; C: *C. glaucum* shells) before (B) and after (A) Pb adsorption.

Biosorbent	Wave number (cm^−1^)		Corresponding functional groups
	B	A	
C	854.814	856.134	CO_3_^2–^
	1082.454	1082.789	C–O
	1445.946	1449.595	CH_2_ , CH_3_
O	1785.548	1786.826	C=O
	690.573	–	Alkanes
	869.763	–	Sulfonates
	1000.891	1012.951	C–O
	1265.504	1242.017	C–N
	1420.036	1418.561	CH_2_, CH_3_
	1647.368	–	C–C
	2919.036	2925.253	C–H
	3362.915	–	OH, NH_2_
	685.885	668.668	Alkanes
	873.156	701.887, 724.624, 776.459, 848.946, 880.824,	Sulfonates
	999.177	945.645	C–O
R	1262.292	1041.086, 1118.305, 1169.983, 1218.037	C–N
	1418.948	1418.502	CH_2_, CH_3_
	1559.329	1464.911	N=O
	1653.375	1623.775, 1653.452	C–C
	2925.660	2919.623	C–H
	3502.807	3118.249	O–H, NH_2_

The chemical composition (weight percentage) of the three biosorbents, before and after the adsorption, based on the results of XRF analyses, are presented in Table 4.

**Table 4:** Chemical composition of (%weight) of the biosorbents (O: *O. mykiss* scales; R: *R. kutum* scales; C: *C. glaucum* shells) before (B) and after (A) Pb adsorption.

Compound/Element	Biosorbent					
	C		R		O	
	B	A	B	A	B	A
Na_2_O	1.421	1.193	ND	ND	1.266	1.162
MgO	1.217	1.087	1.039	0.867	ND	ND
P_2_O_5_	35.373	34.936	24.499	23.739	ND	ND
SO_3_	3.637	3.071	9.452	7.973	0.255	0.171
CaO	58.107	56.048	65.010	63.932	97.635	96.913
Sr	0.245	0.229	ND	ND	0.844	0.775
Pb	ND	3.436	ND	3.489	ND	0.979

*ND* not detectable.

**Figure 5:**
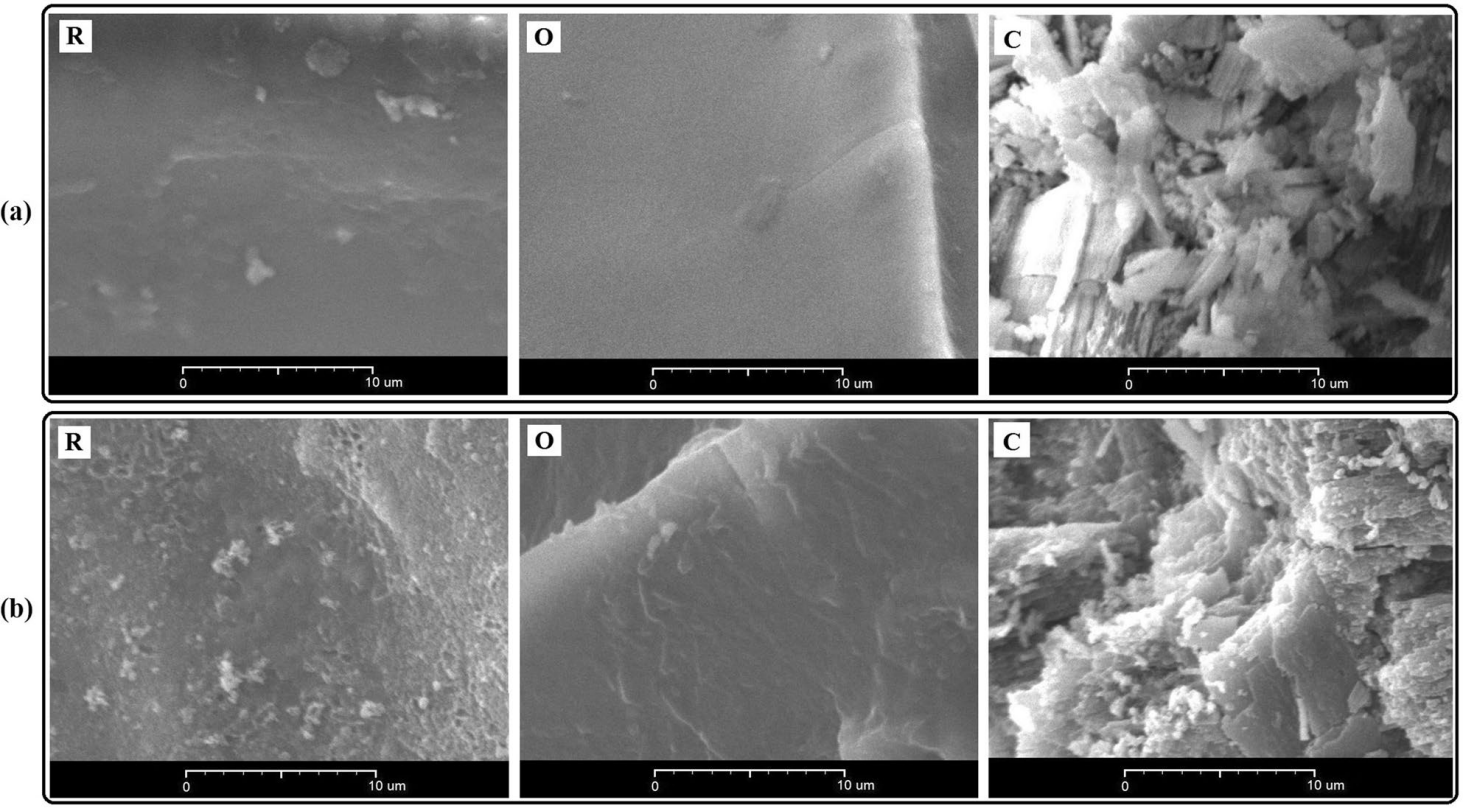
SEM micrographs at 3000 × magnification before (**a**) and after (**b**) Pb biosorption for *C. glaucum* shell (C), *O. mykiss* scale (O), and *R. kutum* scale (R).

**Figure 6:**
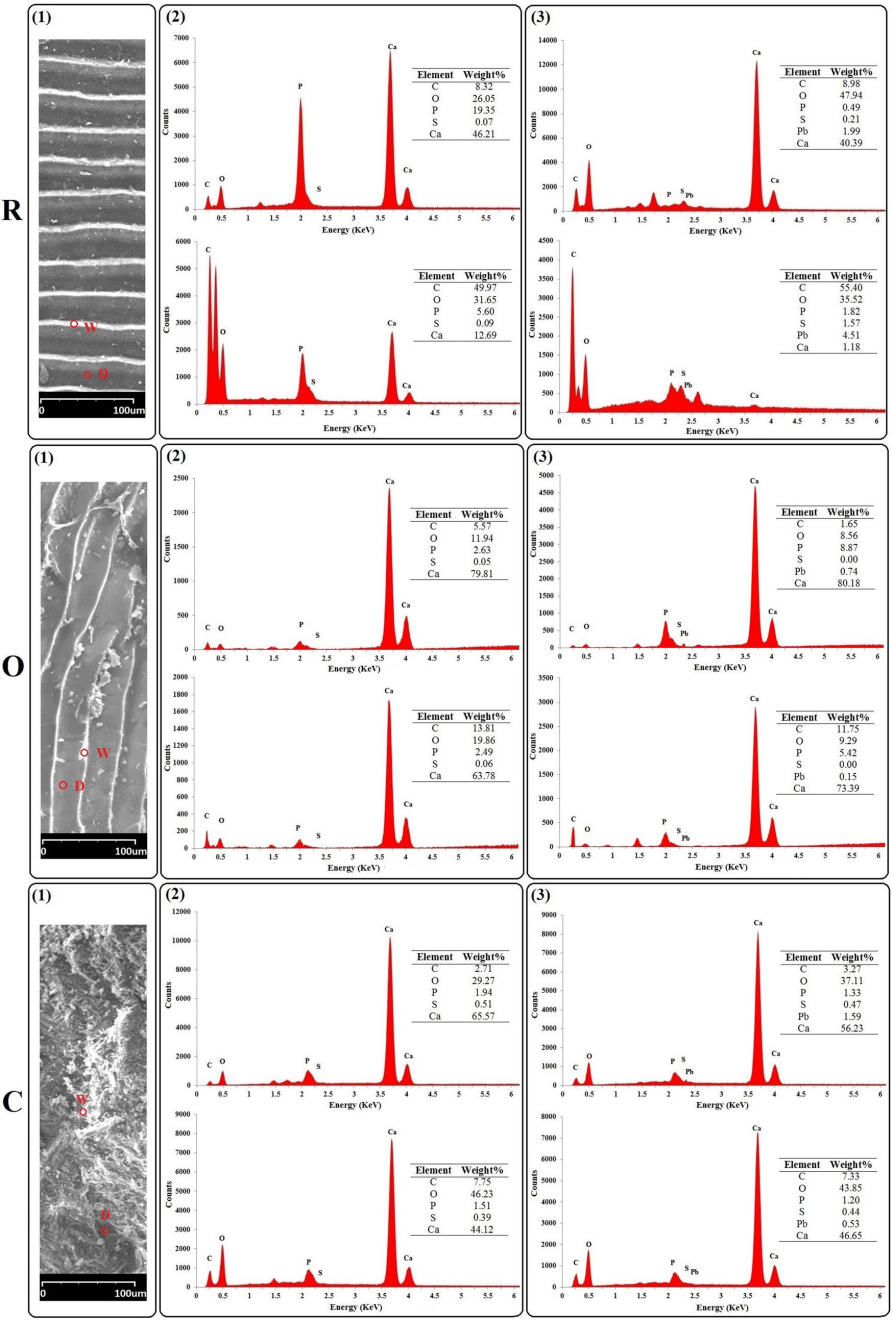
SEM images and corresponding EDX spectra of the three selected biosorbents (R: *R. kutum* scales; O: *O. mykiss* scales; C: *C. glaucum* shells): (1) the selected white (W) and dark (D) spots on the samples (× 200 magnification), (2) EDX spectra of the white (upper) and dark (lower) spots before Pb biosorption, (3) EDX spectra of the white (upper) and dark (lower) spots after Pb biosorption. Insets: Elemental composition (weight percentage).

Figure 5 shows the surface morphologies of the three selected biosorbents before and after the biosorption, characterized using SEM. The EDX spectra of *R. kutum* scales, *O. mykiss* scales and *C. glaucum* shells are presented in Fig. 6. The spectra are depicted for dark and white areas of the biosorbents’ surfaces, before and after the adsorption, along with their elemental composition. In each case, the relevant weight percentages are also presented.

### Adsorption isotherms

**Figure 7:**
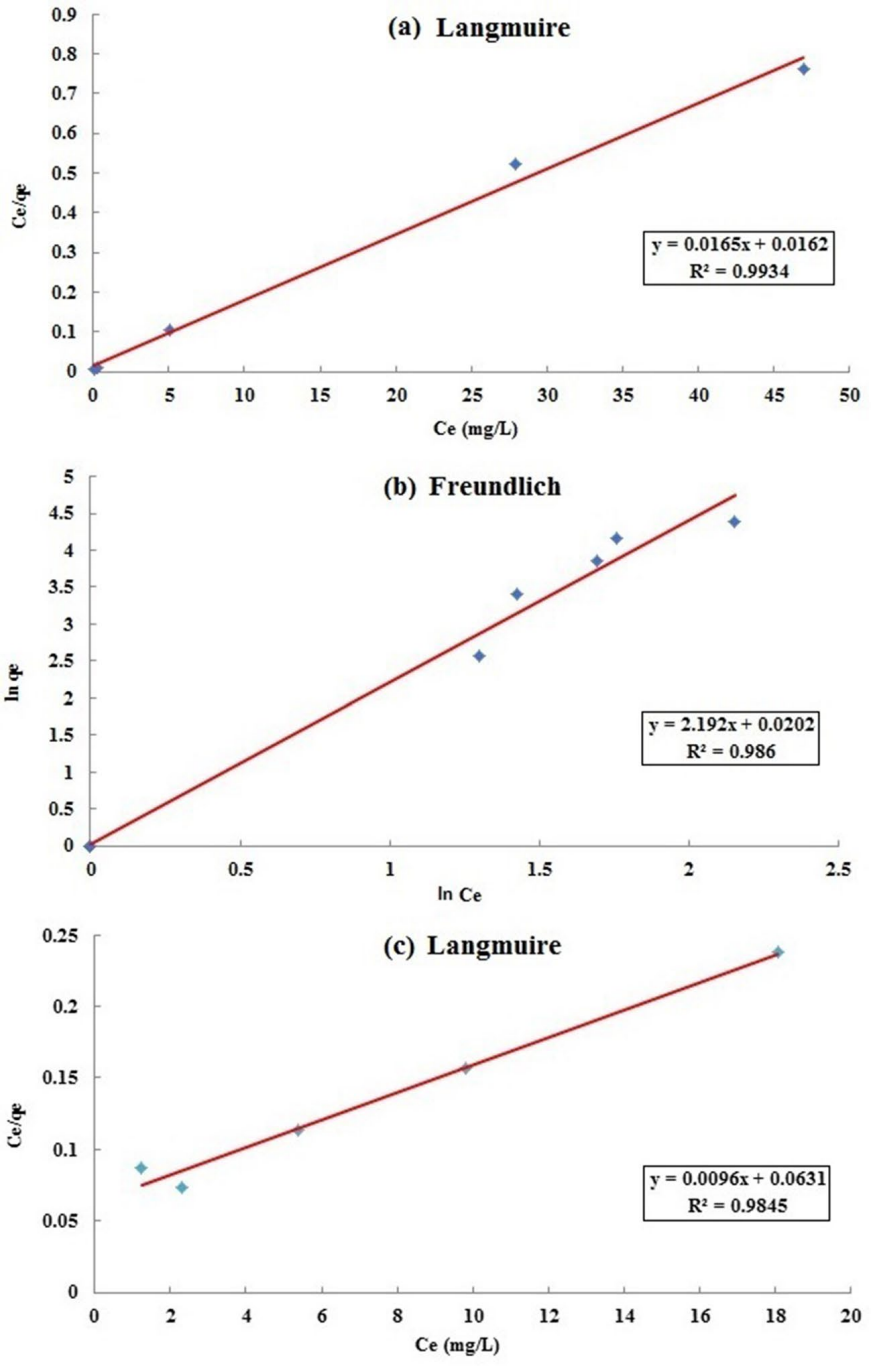
(**a**) Langmuir, (**b**) Freundlich and (**c**) Langmuir isotherm plots for lead adsorption onto *R. kutum* scale, *C. glaucum* shell and *O. mykiss* scale, respectively.

The values of parameters associated with equilibrium isotherms of Pb removal onto the three biosorbents are shown in Table 5. Figure 7 shows the isotherm models that are fitted to our experimental data.

**Table 5:** Parameters of Langmuir, Freundlich, Temkin and Dubinin-Radushkevich (D-R) isotherm models for the adsorption of lead onto the three selected biosorbents (O: *O. mykiss* scales; R: *R. kutum* scales; C: *C. glaucum* shells).

Biosorbents	Isotherm model	Parameter	Value
R	Langmuir	q_max_ (mg/L)	60.61
		K_L_ (L/g)	1.019
		R_L_ range	0.01–0.03
		R^2^	0.9934
	Freunlich	n	2.67
		K_f_ (L/g)	7.6722
		R^2^	0.1844
	Temkin	A_T_ (L/g)	12.66
		B_T_	297.964
		R^2^	0.5062
	D-R	q_max_	131.7648
		K	0.0048
		R^2^	0.8761
C	Langmuir	q_max_ (mg/L)	40.82
		K_L_ (L/g)	0.09
		R_L_ range	0.07–0.62
		R^2^	0.3874
	Freunlich	n	0.46
		K_f_ (L/g)	1.0204
		R^2^	0.9860
	Temkin	A_T_ (L/g)	1.13
		B_T_	76.801
		R^2^	0.8334
	D-R	q_max_	6.6004
		K	0.0002
		R^2^	0.7121
O	Langmuir	q_max_ (mg/L)	104.17
		K_L_ (L/g)	0.152
		R_L_ range	0.04–0.18
		R^2^	0.9845
	Freunlich	n	0.68
		K_f_ (L/g)	2.3396
		R^2^	0.7386
	Temkin	A_T_ (L/g)	1.13
		B_T_	95.306
		R^2^	0.9835
	D-R	q_max_	35.2419
		K	0.0034
		R^2^	0.9377

## Discussion

### Data fitting to the model and ANOVA

Considering models 1–3, the values coefficients of determination (i.e. 0.93, 0.86 and 0.87 for PbR, PbO and PbC, respectively) indicated a good fit between predicted values and the experimental data points. For a good model fit R^2^, should be more than 0.8. In general, the closer the R^2^ value is to 1.00 indicating the better fitting and more suitable model for the prediction of the response variables. In all the responses, differences between predicted R^2^ and adjusted R^2^ were less than 0.2 (Table 2), which indicates reasonable agreement between regression coefficients. According to the Table, the AP ratios for all the responses are considerably greater than 4, which describe good model discrimination. Normally the ratio greater than 4 is desirable, for the models to be used effectively. In all the responses, in comparison with the other models, the linear model (for PbC) showing lower PRESS value (the smaller the PRESS value, the better the model’s predictive ability). The low SD and CV values indicate the high precision and reliability of the experiments. According to Table 2, SD values were 0.30, 0.41 and 0.21, whereas CV values were 3.96, 5.66 and 3.50 for PbR, PbO and PbC, respectively. As a general rule, CV should not be higher than ten percent. The CV values calculated in this study were much lower than the limit, indicating high precision of the conducted experiments. The low values of CV showed that the variabilities between the predicted and observed values are low and were indicative of high reliability of the experiments^51, 55–58^.

Considering the above mentioned points, all the selected models have high R^2^ value, significant F-value, a non-significant lack-of-fit *p*-value, desirable AP values and low SD and CV. The results confirm that the responses can be predicted with high reliability. Hence, the models can be applied for predictive purposes.

### Effects of factors on the responses

#### Effects of main variables

##### Significance of the effects

Table 1 can be used to determine which factors significantly affect each response^59^. According to the table, factors biosorbent dosage, initial concentration and pH were significant for all the responses (*p* < 0.05), whereas biosorbent size was not significant for any of the responses. The contact time and temperature were significant only for PbR and PbC, respectively. The salinity was significant for the response variables PbR and PbC.

##### Order of the effects

The relative effects of significant factors on the responses were determined by evaluating the *p*-values and F-ratios (Table 1). The parameter with the lowest *p*-value and the highest F-ratio shows the greatest impact on the response variables^60^. As mentioned previously, the relative influences of the parameters on the response variables may also be deduced from the perturbation plots (Fig. 2). In a perturbation plot, when the variable produces a steep slope or curvature, then the response variable is sensitive to that parameter, while a relatively flat line shows insensitivity to change in that particular variable^61, 62^. Furthermore, the relative effects can also be distinguished by comparing the coefficients of the factors in the regression models. To evaluate the relative effect of the independent variables, the coefficients calculated in the regression Eqs. (1, 2 and 3) can be directly compared^63^. Description of the order of effects of the studied factors on each of the three response variables are provided separately below:

*PbR*: The initial concentration had the highest F-ratio and the lowest *p*-value (301.13 and < 0.0001, respectively). Hence, this factor had the greatest effect on PbR, followed by biosorbent dosage, pH, salinity and contact time (Table 1). The similar results can be obtained from Fig. 2a. The perturbation plot clearly shows that of the five significant independent variables, biosorbent dosage and initial concentration affect the value of PbR more than the others. With regards to the values of the model coefficients in Eq. 1, a similar decreasing order of effects (with the relevant coefficients) was also observed as follows: initial concentration (1.07), biosorbent dosage (0.7235), pH (0.6499), salinity (0.2312) and contact time (0.1768).

*PbO*: According to Table 1, the factor of initial concentration had the highest F-ratio and the lowest *p*-value (169.00 and < 0.0001, respectively). Hence, this factor produced the highest effect on the response, followed by biosorbent dosage and pH. Figure 2b clearly shows that initial concentration has the main and the major effect on PbO followed by biosorbent dosage and pH, which have the medium and low effects on the response, respectively. Considering the regression coefficients in Eq. 2, among the three significant factors, the highest and lowest effects on PbO were for initial concentration and pH, respectively.

*PbC:* From the perturbation plot (Fig. 2c), the following sequence of relative effects of the factors on PbC can be inferred: initial concentration > salinity > pH > biosorbent dosage > temperature. The steep slopes in opposite directions for initial concentration and salinity are quite clear. The significantly lower slopes for pH, biosorbent dosage and temperature show less sensitivity of PbC to changes in these factors. These results are consistent with those presented in Table 1 and Eq. 3. As can be seen, the factors initial concentration and salinity indicated the highest F-ratios and the regression coefficients and the lowest p-values.

##### Positive and negative effects

The regression equations (Eqs. 1, 2 and 3) as well as the perturbation plots (Fig. 2) were used to investigate whether the effect of each factor on the responses is positive or negative. In the equations, negative and positive signs before each term show antagonistic and synergistic effects on the response, respectively^64^. The significant negative and positive effects on each response variable are described below:

*PbR*: As can be seen in Fig. 2a and Eq. 1, the increase in the factors initial concentration and contact time has positive effect on PbR. On the other hand, the increase in biosorbent dosage has negative effect on PbR. It can be noticed from the figure that the factors pH and salinity have also the same effect, but less strong.

*PbO*: It is observed from Fig. 2b and Eq. 2 that the PbO increases with increasing initial concentration and decreasing biosorbent dosage and pH.

*PbC*: Fig. 2c and Eq. 3 depict that with increase in salinity and temperature reduction in PbC was observed (negative effect), while factors initial concentration, pH and biosorbent dosage have a positive effect on the response variable.

In order to simplify the comparisons, all of the above-mentioned descriptions about the significance of the effects, the relative effects of significant factors, and the positive or negative effects are summarized in the Table 6.

**Table 6:** Order of the significant effects of factors on the responses. Minus and plus signs indicate negative and positive, respectively.

Responses	Factors								
PbR	E^+^	>	A^–^	>	F^–^	>	G^–^	>	C^+^
PbO	E^+^	>	A^–^	>	F^–^				
PbC	E^+^	>	G^-^	>	F^+^	>	A^+^	>	D^−^

PbR: Concentration of Pb adsorbed by scales of *Rutilus kutum*; PbO: Concentration of Pb adsorbed by scales of *Oncorhynchus mykiss*; PbC: Concentration of Pb adsorbed by shells of *Cerastoderma glaucum*; A: Biosorbent dosage; C: Contact time; D: Temperature; E: Initial concentration; F: pH; G: Salinity.

#### Interaction between influencing factors

The response surface and contour plots (Fig. 3) are very useful to see the interaction effects of the parameters on the response variables. In general, the shape of the contour plot indicates the natures and extents of the interactions between parameters. A circular contour plot shows that the mutual interactions between corresponding variables are insignificant. In contrast, elliptical or distorted plots are evidence of significant interactions^59, 65–67^. For each response variable, only the significant interactions (based on Table 1) are shown and described separately below. The elliptical contour shapes in the figures confirm that all the mutual interactions are significant.

*PbR*: Fig. 3a depicts that with increase in pH reduction in PbR was observed, but it was observed that with increment in biosorbent dosage, PbR was decreased. The maximum PbR (7655 ppm) occurred at biosorbent dosage of 0.1 g/L and pH 5.5, while the PbR (493 ppm) was minimal at biosorbent dosage of 0.3 g/L and pH 7. Figure 3b shows that with lower pH and salinity, higher PbR was observed. The PbR was maximal (3009 ppm) at salinity of 0.2 ppt and pH 5.5, while the minimum PbR (493 ppm) was observed at salinity of 10 ppt and pH 7. At higher initial concentration and lower temperature, higher PbR was observed (Fig. 3c). The maximum PbR (8376 ppm) occurred at initial concentration of 100 ppm and temperature of 20 °C, while the PbR (414 ppm) was minimum at initial concentration of 30 ppm and temperature of 30 °C.

According to Eq. 1, all the three mentioned mutual interactions had negative effects on the PbR.

*PbO:* With regards to Fig. 3d, the maximum PbO occurred at low salinity and high initial concentration. The PbO was maximal (5355 ppm) at initial concentration of 100 ppm and salinity of 0.2 ppt, while the minimum PbO (310 ppm) was observed at initial concentration and salinity of 30 ppm and 10 ppt, respectively.

According to Eq. 2, interaction between salinity and initial concentration had negative effects on the PbO.

#### Possible causes of the effects

With regards to the results presented in Tables 1 and 6 and Fig. 2, the following explanations can be given about the causes of observed significant effects and comparison with similar studies, separately:

##### Initial concentration

The initial concentration had a positive significant effect on all the three dependent variables. The similar findings were also reported on lead biosorption by fish scales and bivalve shells^4, 39^. This could be arisen from the fact that the initial concentration actually plays the role of the driving force required to control the resistance of the mass transfer of metal ions between the aqueous phase and the surface of the sorbents, so higher initial concentrations of metal ions may increase their adsorption. Moreover, with increasing initial Pb ions concentration, higher interaction between the metal ions and the biosorbents, and consequently enhancing the availability of the binding sites on the surface of the biosorbents, could be expected^30, 48, 68^.

##### Biosorbent dosage

It seems that in the present study, the selected range of the biosorbents dosage for the response variables PbR and PbO is higher than the equilibrium levels (i.e. maximum adsorption capacity of the biosorbents with a certain absorbate), while in terms of PbC the reverse trend could be observed. Therefore, higher uptake at low biosorbent concentrations for PbR and PbO could be due to availability of lower number of Pb ions per unit mass of the biosorbents. It can also be relevant to aggregation of the biosorbents particles at higher concentrations, thereby lead to a decline in the surface area of adsorbent and also an increase in the diffusion path length^11, 39^. The trend observed for PbC is possibly due to the availability of more functional groups (adsorption sites) along with the increase of the biosorbent dosage. Similar findings had been reported by other studies as well^36, 69, 70^.

##### pH

Generally, the pH of a solution is one of the most effective environmental parameters for adsorption of heavy metal ions because it might affect strongly the degree of ionization and adsorption sites on the sorbent surface during the biosorption process^36, 48, 71^. In the present research, as was largely expectable, pH was found to be one of the important parameters affecting the adsorption of Pb by the studied biosorbents (Table 2).

The findings of several similar researches on the influences of a wider range of pH (3–7) on the metal sorption process of various biosorbents show that in most cases, with the gradual increment of pH, the following specific trends can be observed: (a) strong positive influences: with an increase in pH, there is an increase in ligands with negative charges which results in increase binding of positively charged ions such as Pb^2+^ via the mechanism of ion exchange^14, 47, 72^, (b) slight positive influences: at higher pH, the reduction in adsorption is possibly due to the abundance of OH^−^ ions, causing increased hindrance to diffusion of organics contributing to the metal ions. The main reason for the small increment in metal removal may be that the adsorption sites are no more influenced by the pH change^71, 72^, (c) negative influences: some more increase in pH usually leads to precipitation of the hydroxide form of the metals ions; therefore true adsorption would not be feasible; thus a decline in the percentage of metal ions removal could be observed^5, 39^.

The results of the present study in terms of scales of the two fish species are consistent with those obtained by some other researchers, e.g. El-Sheikh and Sweileh^73^; Bajić et al.6 and Zayadi and Othman^4^ that explained the Pb biosorption capacity of fish scales decreases gradually with increasing pH value of the solution, in the pH range approximately similar to our study. The possible reason for this trend is explained above (regarding the negative effect of pH). The observed reverse trend in terms of PbC could be attributed to the fact that the interaction between the functional groups of the biosorbent and the heavy metal ions is dependent upon nature of the surface of biosorbent and chemistry of the biosorbate solution, which in turn depends on the pH of the solution^74, 75^. For this reason, in the current study, the maximum biosorption for the scales of both fish species (PbR and PbO) occurred similarly at pH 5.5, while that for the bivalve shells (PbC) was found at pH 7.

##### Salinity

PbR and PbC were significantly and negatively affected by the salinity, but no significant effect could be observed on PbO. Generally, salinity is an important parameter in the biosorption process because the existence of the electrolyte ions in an aqueous environment will cause changes in adsorbate activities, and sorbent surface charge by electrostatic (Coulomb) force^76^. So far, no previous study has been performed on the influence of this parameter on biosorption of metals using mollusk shells and fish scales. However, according to the results of some researches in which other adosorbents have been applied, it can be inferred that the increase in the biosorptive capacity with decreaing salinity is likely because of the fact that at lower sodium-to-lead ratios, the less competition for binding sites between sodium and lead ions could be occurred, and vice versa^77, 78^.

##### Contact time

PbR was the only response variable that significantly affected by the contact time. The observed positive effect was also reported by Zayadi and Othman^4^, who found that an increase in contact time leads to increase in Pb removal from aqueous environment by fish scales as biosorbent. This implies that initially, the biosorbent contains a higher number of binding sites for the binding of Pb. In the studies that the range of contact time was wider compared to that of current research, after a lapse of some time, depending on the biosorbent and the solution environment, the number of unoccupied sites decreased and gradually became saturated^5, 39, 79^.

##### Temperature

Of the three response variables, only PbC was significantly affected by temperature. This negative effect has also been observed in some other studies concerning the use of mollusk shells as biosorbents for metals removal from aqueous solutions (e.g. Shahzad et al.^5^; Weerasooriyagedra and Anand Kumar^40^). Since, it is believed that sorption reactions are normally exothermic and, therefore, the decrease in biosorption capacity at higher temperatures likely occurs due to desorption caused by an increase in the available thermal energy. In other words, higher temperature induces higher mobility of the adsorbate causing desorption^5, 80^. It is noticeable that, based on the results of various related studies, the effect of temperature on the biosorption process shows different and contradictory behaviors^81^. The positive effect of temperature on the process, which has been observed in most similar studies, could be attributed to either higher affinity of sites for heavy metal ions or many more binding sites being available on the relevant particle surface at higher temperatures^15, 39^.

### Characterization of the biosorbents

#### FT-IR analysis

Various functional groups play a significant role in the adsorption processes of metal ions as well as the sorption potential of adsorbents. The number and type of functional groups located on the surface of different sorbents affect the adsorption mechanisms^82, 83^. The functional groups actually provide sites for the effective adsorption of heavy metals on the adsorbent surface, and their adsorption potential can be influenced by a relatively wide range of factors^84^. The results of this study showed that the three investigated biosorbents consist of a variety of functional groups capable of binding heavy metal ions. The complexation of functional groups with Pb^2+^ changes their chemical environment and thus leads to shifts or disappearance of the peaks in the FTIR spectra. In other words, the peak shifts and disappearances observed after the adsorption can be considered strong evidence for adsorption of Pb^2+^on the surface of the biosorbents^85, 86^. The presence of similar functional groups as well as their shifts after Pb adsorption on the surface of different aquatics-based biosorbents were also reported by some other researchers^4–6, 27, 29, 39, 87^.

#### XRF analysis

The XRF results (Table 4) showed that the chemical composition of *O. mykiss and R. kutum* scales was dominated by CaO and P_2_O_5_, whereas the contents of the other elements were rather low. In the case of *C. glaucum* shells, calcium oxide was also the main constituent, but P_2_O_5_was not detectable. These findings are in concordance with the results reported by several other researchers who analyzed the chemical composition of other aquatics-based sorbents^4, 15, 29, 39, 70, 88^. It was observed that after Pb adsorption, ion percentage of other elements was decreased. In this case other elements may be involved in ion exchange process with the lead ions.

#### SEM and EDX analysis

The micrographs revealed that the surfaces of the scales of the two species were relatively homogeneous and smooth, but in the case of *C. glaucum* shell an uneven and heterogeneous surface could be observed^89, 90^. Generally, the differences in adsorption capacity of different types of biosorbents depend on a number of factors, among which the surface morphology, composition and porosity are especially important^91–93^. Therefore, the observed differences in the surface microstructures of the three biosorbents (Fig. 5a) can be effective in their different adsorption capacities. The observed significant changes in the morphological characteristics of the biosorbents and some precipitation on their surfaces after the adsorption (Fig. 5b) are evidence of the potential of the biosorbents for adsorption and removal of the metal ions from aqueous solutions^94^. The results of several other studies have also shown that the surface morphology of the adsorbents of aquatic origin has changed after the adsorption of some heavy metals (e.g. Villanueva-Espinosa et al.^95^; Prabu et al.^27^; Muthulakshmi Andal et al.^84^; Yousefi et al.^1^; Xu et al.^15^, Dulla et al.^96^, El-Naggar et al.^97^). Generally, the emergence of post-biosorption peaks that characterize Pb (Fig. 6) indicates the binding of the metal ions to the sorbents surfaces. Hence, with regards to the results of EDX analysis, there are strong and logical reasons for adsorption of Pb ions on the investigated biosorbents^98^. The concentration of the adsorbed elements is directly related to the height of the corresponding EDX peaks^99^. The SEM analyses showed the existence of two regions, i.e., dark and white areas. The dark region is mainly composed of proteins containing large amounts of carbon, oxygen, and sulfur, whereas the white region is mainly consists of inorganic components, including high amounts of calcium and phosphorus^4, 95^. The difference between the two regions can also be deduced from the elemental composition of the adsorbent surface, as shown in the insets of the figures. With regards to Fig. 6, in the case of *C. glaucum* shells and *O. mykiss* scales, the greater Pb adsorption was detected in the white region, whereas in the case of *R. kutum* scales the darker area showed the higher adsorption capacity. The different adsorption values observed in the white and dark regions are probably mainly caused by the differences in number and type of the functional groups, microstructure, surface morphology and chemical nature of the sorbents^100–102^.

#### Sorption isotherms

According to values of regression coefficients (R^2^), the Langmuir isotherm showed the best fitted values for *R. kutum* scale (R^2^ = 0.9934) and *O. mykiss* scale (R^2^ = 0.9845) (Table 5 and Fig. 7). Therefore, it can be opined that the two biosorbents may have homogeneous surfaces and monolayer adsorption^103^. The separation factor (R_L_) values were between 0 and 1, indicating a favorable adsorption of Pb onto the two biosorbents^104^. While the Freundlich model was suitable for the equilibrium isotherm of *C. glaucum* shell (R^2^ = 0.9860). Contrary to Langmuir isotherm, the Freundlich isotherm is applicable to heterogeneous surfaces and multilayer adsorption^105^.

#### Comparison with other aquatics-based biosorbents

The biosorption capacities of the three studied biosorbents in the present study in comparison with those of other biosorbents reported is shown in Table 7. These data show that the sorption capacities of the three biosorbents are comparable to those of other sorbents reported in the literature (within the range of 0.86 and 248.00 mg/g for fish scales and brown seaweed, respectively).

**Table 7:** The comparison of biosorption capacity for lead with various biosorbents.

Biosorbent	Max sorption capacity (mg/g)	Reference
Fish scales (*Rutilus kutum*)	24.26	Present study
Fish scales (*Oncorhynchus mykiss*)	14.39	Present study
Bivalve mollusk shell (*Cerastoderma glaucum*(	1.29	Present study
Fish scales (*Labeo rohita*)	196.80	Nadeem et al.^106^
Fish scales (*Genyonemus lineatus*)	0.86	Onwordi et al.^107^
Fish scales (*Cyprinus carpio*)	62.5	Bajić et al.^6^
Fish fins (*Catla catla*)	3.00	Gupta et al.^108^
Bivalve mollusk shell (*Anodontoides ferussacianus*)	155.04	Shahzad et al.^5^
Cockle shell	24.66	Ayodele and Adekola^39^
Freshwater snail shell (*Melanoides tuberculate*)	0.59	Castañeda et al.^109^
Chitin of shrimp (*Solenocera melantho*)	7.00	Forutan et al.^110^
Marine brown algae (*Cystoseira stricta*)	64.5	Iddou et al.^111^
Green seaweed (*Ulva lactuca*)	2.25	Sari and Tuzen^112^
Brown seaweed (*Cystoseira baccata*)	124.00	Lodeiro et al.^113^
Brown seaweed (*Laminaria japonica*)	248.00	Luo et al.^114^
Aquatic plant (*Hydrilla verticillata*)	2.14	Dileepa Chathuranga et al.^11^
Aquatic plant (*Myriophyllum spicatum*)	55.12	Yan et al.^115^

In general, It should be noted that direct comparison of sorption capacities of different biosorbents listed in the table is difficult due to: (a) the sorbents have been investigated under various preparation and test conditions (including contact time, pH, particle size, metal concentration range, temperature, mixing rate and ….), (b) the methods of pre-treatment and preparation of the biosorbents are not similar in different investigations, and (c) the techniques for determining maximum adsorption capacity (e.g. BBD-designed experiments, Langmuir Isotherm, Freundlich Isotherm, Pseudo-second order kinetics) have been different in various studies. The first and second points show the factors that may play an important role in increasing the adsorption capacity of sorbents for a given heavy metal^26, 116^, and the third point indicates the difference in calculation methods.

With regards to Table 7, a comparison of the three biosorbents studied in the present study shows that the ascending order of the sorption capacity is: the shells of *C. glaucum*, scales of *O. mykiss* and scales of *R. kutum*. Given that the preparation methods and experimental conditions were the same for all three sorbents, these differences in adsorption capacities are probably mostly arose from the differences in surface area, morphology and functional groups^117, 118^. On the other side, according to Regine et al.^119^ the role of the functional groups in biosorption of a given metal by a certain biosorbent is related to several factors, including accessibility of the reactive sites, the number of the sites in the biosorbent, chemical state of the sites (i.e. availability), and affinity between the sites and the particular metal.

## Conclusion

Among the seven studied parameters in this study, the effects of biosorbent dosage, initial concentration and pH on the Pb biosorptive potential of all the three sorbents were significant (*p* < 0.05), while biosorbent size was not significant for any of the response variables. It was found that the initial concentration was the most influential factor, which had positive effect on adsorption capacity of the three biosorbents. The considerable effects of initial concentration on adsorption efficiency of various biosorbents for heavy metals have also been reported by some other researchers (e.g. Osu and Odoemelam^69^; Zayadi and Othman^4^; Dileepa Chathuranga et al.^11^; Ayodele and Adekola^39^; Al-Saeedi et al*.*^36^).

The SEM and EDX analyses confirmed Pb biosorption as obvious changes in the surface morphologies of the sorbents, and as appearance of characteristic peaks in the EDX spectra. The XRF results were also confirmed the presence of Pb on the surface of the biosorbents after the adsorption and implied the probable ion exchange between the positively charged ions on the sorbents with Pb ions. The FTIR results showed that the three investigated biosorbents contained several functional groups that can participate in metal binding. The observed shifts and disappearances of the bands indicated that the functional groups were involved or affected by complexation with Pb^2+^. With regards to the potential roles of functional groups in heavy metals adsorption process^120, 121^, less diversity of the groups on the surface of *C. glaucum* shells compared to the other two biosorbents probably contributes to the lower adsorption capacity of this adsorbent (Table 7). In this regard, it should be noted that, the findings of several related studies investigating the role of functional groups in the adsorption of Pb(II) ions by various adsorbents indicate that among the diverse identified functional groups present on the adsorbents surface, the hydroxyl, amine, sulfonate and carboxyl groups play a significant role^122–127^. The peak shifts of the mentioned functional groups were also observed in the biosorbents studied in the present research, especially for the fish scales (Table 7), which confirms the higher adsorption potential of lead ions by these adsorbents compare to the bivalve shells.

Among the isotherm models tested, the Langmuir model was in the best agreement with the experimental data for both PbR and PbO, whereas the Freundlich model agreed well with the adsorption data of PbC.

Generally, it can be concluded that the investigated biosorbents, especially scales of *O. mykiss* and *R. kutum* can be considered as potential biosorbents for the removal of Pb from aqueous solutions. The biosorbents are promising alternative to the conventional treatment methods due to their low cost, eco-friendliness and easy availability. However, additional studies are recommended to be conducted in this regard to explore: (a) possible roles of various chemical and physical pretreatment methods of the biosorbents in their heavy metal removal efficiency, (b) influences of other factors on the adsorption capacity of the sorbents, (c) efficiency of the biosorbents in the removal of other heavy metals, (d) possibility of reuse the biosorbents, (e) feasibility of using the selected biosorbents at an industrial scale, (f) possibility of using different biosorbents mixtures for enhancement of heavy metals removal efficiency, and (g) maximum adsorption capacity of other aquatics-based sorbents.
